# ML-DSP: Machine Learning with Digital Signal Processing for ultrafast, accurate, and scalable genome classification at all taxonomic levels

**DOI:** 10.1186/s12864-019-5571-y

**Published:** 2019-04-03

**Authors:** Gurjit S. Randhawa, Kathleen A. Hill, Lila Kari

**Affiliations:** 1 Department of Computer Science, University of Western Ontario, London, ON, Canada; 2 Department of Biology, University of Western Ontario, London, ON, Canada; 3 School of Computer Science, University of Waterloo, Waterloo, ON, Canada

**Keywords:** Taxonomic classification, Whole genome analysis, Genomic signature, Alignment-free sequence analysis, Machine learning, Numerical representation of DNA sequences, Digital signal processing, Discrete Fourier transform

## Abstract

**Background:**

Although software tools abound for the comparison, analysis, identification, and classification of genomic sequences, taxonomic classification remains challenging due to the magnitude of the datasets and the intrinsic problems associated with classification. The need exists for an approach and software tool that addresses the limitations of existing alignment-based methods, as well as the challenges of recently proposed alignment-free methods.

**Results:**

We propose a novel combination of supervised **M**achine **L**earning with **D**igital **S**ignal **P**rocessing, resulting in **ML-DSP**: an alignment-free software tool for ultrafast, accurate, and scalable genome classification at all taxonomic levels. We test ML-DSP by classifying 7396 full mitochondrial genomes at various taxonomic levels, from kingdom to genus, with an average classification accuracy of >97*%*.

A quantitative comparison with state-of-the-art classification software tools is performed, on two small benchmark datasets and one large 4322 vertebrate mtDNA genomes dataset. Our results show that ML-DSP overwhelmingly outperforms the alignment-based software MEGA7 (alignment with MUSCLE or CLUSTALW) in terms of processing time, while having comparable classification accuracies for small datasets and superior accuracies for the large dataset. Compared with the alignment-free software FFP (Feature Frequency Profile), ML-DSP has significantly better classification accuracy, and is overall faster.

We also provide preliminary experiments indicating the potential of ML-DSP to be used for other datasets, by classifying 4271 complete dengue virus genomes into subtypes with 100% accuracy, and 4,710 bacterial genomes into phyla with 95.5% accuracy.

Lastly, our analysis shows that the “Purine/Pyrimidine”, “Just-A” and “Real” numerical representations of DNA sequences outperform ten other such numerical representations used in the Digital Signal Processing literature for DNA classification purposes.

**Conclusions:**

Due to its superior classification accuracy, speed, and scalability to large datasets, ML-DSP is highly relevant in the classification of newly discovered organisms, in distinguishing genomic signatures and identifying their mechanistic determinants, and in evaluating genome integrity.

## Background

Of the estimated 8.7 million (±1.3 million) species existing on Earth [1], only around 1.5 million distinct eukaryotes have been catalogued and classified so far [2], leaving 86% of existing species on Earth and 91% of marine species still unclassified. To address the grand challenge of all species identification and classification, a multitude of techniques have been proposed for genomic sequence analysis and comparison. These methods can be broadly classified into alignment-based and alignment-free. Alignment-based methods and software tools are numerous, and include, e.g., MEGA7 [3] with sequence alignment using MUSCLE [4], or CLUSTALW [5, 6]. Though alignment-based methods have been used with significant success for genome classification, they have limitations [7] such as the heavy time/memory computational cost for multiple alignment in multigenome scale sequence data, the need for continuous homologous sequences, and the dependence on a priori assumptions on, e.g., the gap penalty and threshold values for statistical parameters [8]. In addition, with next-generation sequencing (NGS) playing an increasingly important role, it may not always be possible to align many short reads coming from different parts of genomes [9]. To address situations where alignment-based methods fail or are insufficient, alignment-free methods have been proposed [10], including approaches based on Chaos Game Representation of DNA sequences [11–13], random walk [14], graph theory [15], iterated maps [16], information theory [17], category-position-frequency [18], spaced-words frequencies [19], Markov-model [20], thermal melting profiles [21], word analysis [22], among others. Software implementations of alignment-free methods also exist, among them COMET [23], CASTOR [24], SCUEAL [25], REGA [26], KAMERIS [27], and FFP (Feature Frequency Profile) [28]. While alignment-free methods address some of the issues faced by alignment-based methods, [7] identified the following challenges they face:

(i) Lack of software implementation: Most of the existing alignment-free methods are still exploring technical foundations and lack software implementation, which is necessary for methods to be compared on common datasets.
(ii) Use of simulated sequences or very small real world datasets: The majority of the existing alignment-free methods are tested using simulated sequences or very small real-world datasets. This makes it hard for experts to pick one tool over the others.
(iii) Memory overhead: Scalability to multigenome data can cause memory overhead in word-based methods, especially when long *k*-mers are used.

To overcome these challenges, we propose ML-DSP, a novel combination of supervised **M**achine **L**earning with **D**igital **S**ignal **P**rocessing of the input DNA sequences, as a general-purpose alignment-free method and software tool for genomic DNA sequence classification at all taxonomic levels.

The main contribution of ML-DSP is the *feature vector* that we propose to be used by the supervised learning algorithms. Given a genomic DNA sequence, its feature vector consists of the pairwise Pearson Correlation Coefficient (PCC) between (a) the magnitude spectrum of the Discrete Fourier Transform (DFT) of the digital signal obtained from the given sequence by some suitable numerical encoding of the letters *A*, *C*, *G*, *T* into numbers, and (b) the magnitude spectra of the DFT of all the other genomic sequences in the training set. The use of this new feature vector, which has not previously been used in conjunction with machine learning algorithms, allows ML-DSP to significantly outperform existing methods in terms of speed, while achieving an average classification accuracy of >97*%*. This substantial performance improvement allows ML-DSP to scale up and successfully classify much larger datasets than existing studies. Indeed, in contrast with previous benchmark datasets, each comprising less than fifty sequences, this study accurately classifies thousands of genomes from a variety of species: eukaryotic (7396 complete mitochondrial genomes), viral (4271 genomes), and bacterial (4710 genomes). In addition, this study provides the first comprehensive analysis and comparison of all thirteen one-dimensional numerical representations of DNA sequences used in the Genomic Signal Processing (GSP: digital signal processing applied to genomes) literature for classification purposes. We conclude that the “Purine/Pyrimidine (PP)”, “Just-A”, and “Real” numerical representations are the top three performers in terms of classification accuracy of ML-DSP for our main dataset. This is surprising given that these three numerical representations do not appear to contain sufficient biological information for the accuracy attained. For example, the numerical representation “Just-A” (encoding *A* as “1”, and *G*,*C*,*T* as “0”) retains the incidence and spacing for *A*, but not individually for the other three nucleotides.

### Numerical representations of DNA sequences

Digital Signal Processing (DSP) can be employed in the context of comparative genomics because genomic sequences can be numerically represented as discrete numerical sequences and hence treated as digital signals. Several numerical representations of DNA sequences, that use numbers assigned to individual nucleotides, have been proposed in the literature [29], e.g., based on a fixed mapping of each nucleotide to a number, without biological significance; using mappings of nucleotides to numerical values deduced from their physio-chemical properties; or using numerical values deduced from the doublets or codons that the individual nucleotide was part of [29, 30]. In [31, 32] three physio-chemical based representations of DNA sequences (atomic, molecular mass, and Electron-Ion Interaction Potential, EIIP) were considered for genomic analysis, and the authors concluded that the choice of numerical representation did not have any effect on the results. A recent study comparing different numerical representation techniques on a small dataset [33] concluded that multi-dimensional representations (such as Chaos Game Representation) yielded better genomic comparison results than some one-dimensional representations. However, in general there is no agreement on whether or not the choice of numerical representation for DNA sequences makes a difference on the genome comparison results, or on which numerical representations are best suited for analyzing genomic data. We address this issue by providing a comprehensive analysis and comparison of thirteen one-dimensional numerical representations, for suitability in genome analysis.

### Digital signal processing

Following the choice of a suitable numerical representation for DNA sequences, DSP techniques can be applied to the resulting discrete numerical sequences, and the whole process has been termed Genomic Signal Processing (GSP) [30]. DSP techniques have previously been used for DNA sequence comparison, e.g., to distinguish coding regions from non-coding regions [34–36], to align genomic signals for classification of biological sequences [37], for whole genome phylogenetic analysis [38], and to analyze other properties of genomic sequences [39]. In our approach, genomic sequences are represented as discrete numerical sequences, treated as digital signals, transformed via DFT into corresponding magnitude spectra, and compared via Pearson Correlation Coefficient (PCC) to create a pairwise distance matrix.

### Supervised machine learning

Machine learning has been used in small-scale genomic analysis studies [40–42], and classification analyses associated with microarray gene expression data [43–45]. In this vein, ML-DSP focusses on the use of the primary DNA sequence data for taxonomic classification, and is based on a novel combination of supervised machine learning with feature vectors consisting of the pairwise distances between the magnitude spectrum of the DFT obtained from the digital signal generated from a DNA sequence, and the magnitude spectra of the DFT of the digital signals generated from all other sequences in the training set. The taxonomic labels of sequences are provided for training purposes. Six supervised machine learning classifiers (Linear Discriminant, Linear SVM, Quadratic SVM, Fine KNN, Subspace Discriminant, and Subspace KNN) are trained on these pairwise distance vectors, and then used to classify new sequences. Independently, classical MultiDimensional Scaling (MDS) generates a 3D visualization, called Molecular Distance Map (MoDMap) [46], of the interrelationships among all sequences.

For our computational experiments, we used a large dataset of 7396 complete mtDNA sequences, and six different classifiers, to compare one-dimensional numerical representations for DNA sequences used in the literature for classification purposes. For this dataset, we concluded that the “PP”, “Just-A”, and “Real” numerical representations were the best numerical representations. We analyzed the performance of ML-DSP in classifying the aforementioned genomic mtDNA sequences, from the highest level (domain into kingdoms) to lower level (family into genera) taxonomical ranks. The average classification accuracy of ML-DSP was >97*%* when using the “PP”, “Just-A”, and “Real” numerical representations.

To evaluate our method, we compared its performance (accuracy and speed) on three datasets: two previously used small benchmark datasets [47], and a large real world dataset of 4322 complete vertebrate mtDNA sequences. We found that ML-DSP had significantly better accuracy scores than the alignment-free method FFP on all datasets. When compared to the state-of-the-art alignment-based method MEGA7 (with alignment using MUSCLE or CLUSTALW), ML-DSP achieved similar accuracy but superior processing times (2250 to 67,600 times faster) for the small benchmark dataset of 41 mammalian genomes. The contrast in running time was even more extreme for the large dataset of 4322 mtDNA genomes, where ML-DSP took 28 s, while MEGA7(MUSCLE/CLUSTALW) could not complete the computation after 2 h/6 h and had to be terminated.

Lastly, we provide preliminary computational experiments that indicate the potential of ML-DSP to successfully classify viral genomes (4271 complete dengue virus genomes into four subtypes) and bacterial genomes (4710 complete bacterial genomes into three phyla).

## Methods and implementation

The main idea behind ML-DSP is to combine supervised machine learning techniques with digital signal processing, for the purpose of DNA sequence classification. More precisely, for a given set *S*={*S*_1_,*S*_2_,…,*S*_*n*_} of *n* DNA sequences, ML-DSP uses:

- DNA numerical representations to obtain a set *N*={*N*_1_,*N*_2_,…,*N*_*n*_} where *N*_*i*_ is a discrete numerical representation of the sequence *S*_*i*_, 1≤*i*≤*n*.
- Discrete Fourier Transform (DFT) applied to the length-normalized digital signals *N*_*i*_, to obtain the frequency distribution; the magnitude spectrum *M*_*i*_ of this frequency distribution is then obtained.
- Pearson Correlation Coefficient (PCC) to compute the distance matrix of all pairwise distances for each pair of magnitude spectra (*M*_*i*_,*M*_*j*_), where 1≤*i*,*j*≤*n*.
- Supervised Machine Learning classifiers which take the pairwise distance matrix for a set of sequences, together with their respective taxonomic labels, in a training set, and output the taxonomic classification of a new DNA sequence. To measure the performance of such a classifier, we use the 10-fold cross-validation technique.
- Independently, Classical Multidimensional Scaling (MDS) takes the distance matrix as input and returns an (*n*×*q*) coordinate matrix, where *n* is the number of points (each point represents a unique sequence from set *S*) and *q* is the number of dimensions. The first three dimensions are used to display a MoDMap, which is the simultaneous visualization of all points in 3*D*-space.

### DNA numerical representations

To apply digital signal processing techniques to genomic data, genomic sequences are first mapped into discrete numerical representations of genomic sequences, called *genomic signals* [48]. In our analysis of various numerical representations for DNA sequences (Table 1), we considered only 1*D* numerical representations, that is, those which produce a single output numerical sequence, called also *indicator sequence*, for a given input DNA sequence.

**Table 1:** Numerical representations of DNA sequences

#	Representation	Rules	Output for *S*_1_ = *CGAT*
1	Integer	*T*=0, *C*=1, *A*=2, *G*=3	[ 1 3 2 0]
2	Integer (other variant)	*T*=1, *C*=2, *A*=3, *G*=4	[ 2 4 3 1]
3	Real	*T*=−1.5, *C*=0.5, *A*=1.5, *G*=−0.5	[ 0.5 −0.5 1.5 −1.5]
4	Atomic	*T*=6, *C*=58, *A*=70, *G*=78	[ 58 78 70 6]
5	EIIP (electron-ion interaction potential)	*T*=0.1335, *C*=0.1340, *A*=0.1260, *G*=0.0806	[ 0.1340 0.8060 0.1260 0.1335]
6	PP (purine/pyrimidine)	*T*/*C*=1, *A*/*G*=−1	[ 1 −1 −1 1]
7	Paired numeric	*T*/*A*=1, *C*/*G*=−1	[ −1 −1 1 1]
8	Nearest-neighbor based doublet	0−15 for all possible doublets	[ 14 8 1 7]
9	Codon	0−63 for all possible 64 Codons	[ 2 35 22 44]
10	Just-A	*A*=1, *rest*=0	[ 0 0 1 0]
11	Just-C	*C*=1, *rest*=0	[ 1 0 0 0]
12	Just-G	*G*=1, *rest*=0	[ 0 1 0 0]
13	Just-T	*T*=1, *rest*=0	[ 0 0 0 1]

Numerical representations of DNA sequences analyzed for usability in genomic classification with ML-DSP. The second column lists the numerical representation name, the third column describes the rule it uses, and the fourth is the output of this rule for the input DNA sequence *S*_1_=*CGAT*. For the nearest-neighbor based doublet representation and codon representation, the DNA sequence is considered to be wrapped (the last position is followed by the first)

We did not consider other numerical representations, such as binary [29], or nearest dissimilar nucleotide [49], because those generate four numerical sequences for each genomic sequence, and would thus not be scalable to classifications of thousands of complete genomes.

### Discrete Fourier Transform (DFT)

Our alignment-free classification method of DNA sequences makes use of the DFT magnitude spectra of the discrete numerical sequences (discrete digital signals) that represent DNA sequences. In some sense, these DFT magnitude spectra reflect the nucleotide distribution of the originating DNA sequences.

To start with, assuming that all input DNA sequences have the same length *p*, for each DNA sequence *S*_*i*_=(*S*_*i*_(0),*S*_*i*_(1),…,*S*_*i*_(*p*−1)), in the input dataset, where 1≤*i*≤*n*, *S*_*i*_(*k*)∈{*A*,*C*,*G*,*T*}, 0≤*k*≤*p*−1, we calculate its corresponding discrete numerical representation (discrete digital signal) *N*_*i*_ defined as

$$N_{i} = \left(f\left(S_{i}(0)\right), f\left(S_{i}(1)\right), \dots, f\left(S_{i}(p-1)\right)\right) $$

where, for each 0≤*k*≤*p*−1, the quantity *f*(*S*_*i*_(*k*)) is the value under the numerical representation *f* of the nucleotide in the position *k* of the DNA sequence *S*_*i*_.

Then, the DFT of the signal *N*_*i*_ is computed as the vector *F*_*i*_ where, for 0≤*k*≤*p*−1 we have

1 $$\begin{array}{@{}rcl@{}} F_{i}(k) = \sum\limits_{j=0}^{p-1} f\left(S_{i}(j)\right) \cdot e^{(-2\pi i/p)kj} \end{array} $$

The magnitude vector corresponding to the signal *N*_*i*_ can now be defined as the vector *M*_*i*_ where, for each 0≤*k*≤*p*−1, the value *M*_*i*_(*k*) is the absolute value of *F*_*i*_(*k*), that is, *M*_*i*_(*k*)=|*F*_*i*_(*k*)|. The magnitude vector *M*_*i*_ is also called the magnitude spectrum of the digital signal *N*_*i*_ and, by extension, of the DNA sequence *S*_*i*_. For example, if the numerical representation *f* is Integer (row 1 in Table 1), then for the sequence *S*_1_=*CGAT*, the corresponding numerical representation is *N*_1_=(1,3,2,0), the result of applying DFT is *F*_1_=(6, −1−3*i*, 0, −1+3*i*) and its magnitude spectrum is *M*_1_=(6, 3.1623, 0, 3.1623).

**Fig. 1:**
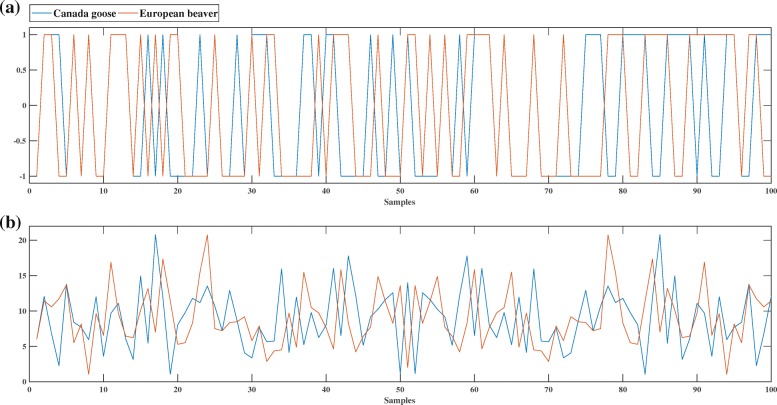
Canada goose (blue) vs European beaver (red): comparison of the DFT magnitude spectra of the first 100 bp of their mtDNA genomes (**a**): Graphical illustration of the discrete digital signals of the respective DNA sequences, obtained using the “PP” representation. (**b**): DFT magnitude spectra of the signals in (**a**)

**Fig. 2:**
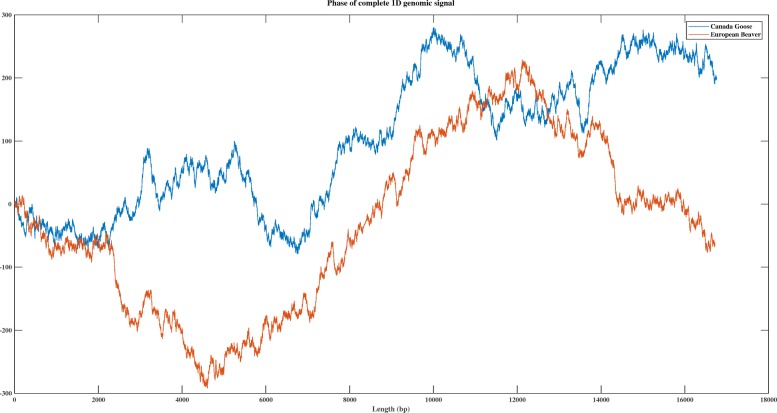
Canada goose (blue, 16,760 bp) vs. European beaver (red, 16,722 bp) - comparison between the DFT phase spectra of their full mtDNA genomes

Figure 1a shows the discrete digital signal (using the “PP” numerical representation, row 6 of Table 1) of the DNA sequence consisting of the first 100 bp of the mtDNA genome of *Branta canadensis* (Canada goose, NCBI accession number *NC*_007011.1), and of the DNA sequence consisting of the first 100 bp of the mtDNA genome of *Castor fiber* (European beaver; NCBI accession number *NC*_028625.1). Figure 1b shows the DFT magnitude spectra of the same two signals/sequences. As can be seen in Fig. 1b, these mtDNA sequences exhibit different DFT magnitude spectrum patterns, and this can be used to distinguish them computationally by using. e.g., the Pearson Correlation Coefficient, as described in the next subsection. Other techniques have also been used for genome similarity analysis, for example comparing the phase spectra of the DFT of digital signals of full mtDNA genomes, as seen in Fig. 2 and [50, 51].

Note that, with the exception of the example in Fig. 1, all of the computational experiments in this paper use full genomes.

### Pearson Correlation Coefficient (PCC)

Consider two variables *X* and *Y* (here *X* and *Y* are the magnitude spectra *M*_*i*_ and *M*_*j*_ of two signals), each of length *p*, that is, *X*={*X*_0_,…,*X*_*p*−1_} and *Y*={*Y*_0_,…,*Y*_*p*−1_}. The Pearson Correlation Coefficient *r*_*XY*_ between *X* and *Y* is the ratio of their covariance (measure of how much *X* and *Y* vary together) to the product of their standard deviations [52, 53], that is,

2 $$\begin{array}{@{}rcl@{}}  r_{XY} = \frac{\sigma_{XY}}{\sigma_{X} \sigma_{Y}} \end{array} $$

where the covariance of *X* and *Y* is $\sigma _{XY}= \sum _{i=0}^{p-1} \left (X_{i}-\overline {X}\right)\left (Y_{i}-\overline {Y}\right)/(p-1)$, and the standard deviation is $\sigma _{X} = \sqrt {\sum _{i=0}^{p-1} \left (X_{i}-\overline {X}\right)^{2}/(p-1)}$, and similarly for *σ*_*Y*_, where the average is defined as $\overline {X} = \left (\sum _{i=0}^{p-1} X_{i}\right)/p$ and similarly for *Y*. Now the formula for the Pearson Correlation Coefficient becomes

3 $$\begin{array}{@{}rcl@{}}  r_{XY} = \frac{\sum_{i=0}^{p-1} \left(X_{i}-\overline{X}\right)\left(Y_{i}-\overline{Y}\right)}{\sqrt{\sum_{i=0}^{p-1} \left(X_{i}-\overline{X}\right)^{2}} \times \sqrt{\sum_{i=0}^{p-1} \left(Y_{i}-\overline{Y}\right)^{2}}} \end{array} $$

The Pearson Correlation Coefficient between *X* and *Y* is a measure of their linear correlation, and has a value between +1 (total positive linear correlation) and −1 (total negative linear correlation); 0 is no linear correlation. We normalized the results, by taking (1−*r*_*XY*_)/2, to obtain distance values between 0 and 1 (value 0 for identical signals, and 1 for negatively correlated signals). For our data sets, the PCC values between any two digital signals of DNA sequences ranged between 0 and 0.6.

For each pairwise distance calculation, the Pearson Correlation Coefficient requires the input variables (that is, the magnitude spectra of the two sequences) to have the same length. The length of a magnitude spectrum is equal to the length of corresponding numerical digital signal, which in turn is equal to the length of the originating DNA sequence. Given that genome sequences are typically of different lengths, it follows that their corresponding digital signals need to be length-normalized, if we are to be able to use the Pearson Correlation Coefficient. Hoang et al. avoided normalization and considered only the first few mathematical moments constructed from the power spectra for comparison, after applying DFT [54]. The limitation of this method is that one loses information that may be necessary for a meaningful comparison. This is especially important when the genomes compared are very similar to each other.

Different methods for length-normalizing digital signals were tested: down-sampling [55], up-sampling to the maximum length using zero padding [30], even scaling extension [56], periodic extension, symmetric padding, or anti-symmetric padding [57]. For example, zero-padding, which adds zeroes to all of the sequences shorter than the maximum length, was used in [30], e.g., for taxonomic classifications of ribosomal S18 subunit genes from twelve organisms. While this method may work for datasets of sequences of similar lengths, it is not suitable for datasets of sequences of very different lengths (our study: fungi mtDNA genomes dataset - 1364 bp to 235,849 bp; plant mtDNA genomes dataset - 12,998 bp to 1,999,595 bp; protist mtDNA genomes dataset - 5882 bp to 77,356 bp). In such cases, zero-padding acts as a tag and may lead to inadvertent classification of sequences based on their length rather than based on their sequence composition. Thus, we employed instead anti-symmetric padding, whereby, starting from the last position of the signal, boundary values are replicated in an anti-symmetric manner. We also considered two possible ways of employing anti-symmetric padding: normalization to the maximum length (where shorter sequences are extended to the maximum sequence length by anti-symmetric padding) vs. normalization to the median length (where shorter sequences are extended by anti-symmetric padding to the median length, while longer sequences are truncated after the median length).

### Supervised machine learning

In this paper we used the Linear discriminant, Linear SVM, Quadratic SVM, Fine KNN, Subspace discriminant and Subspace KNN classifiers from the Classification Learner application of MATLAB (Statistics and Machine Learning Toolbox). The default MATLAB parameters were used.

To assess the performance of the classifiers, we used 10-fold cross validation. In this approach, the dataset is randomly partitioned into 10 equal-size subsets. The classifier is trained using 9 of the subsets, and the accuracy of its prediction is tested on the remaining subset. As part of the supervised learning, taxonomic labels are supplied for the DNA sequences in the 9 subsets used for training. The process is repeated 10 times, and the accuracy score of the classifier is then computed as the average of the accuracies obtained in the 10 separate experiments. The standard algorithms were modified so that no information about sequences in the testing set (that is, no distance matrix entries containing distances to/from any sequence in the testing set to any other sequence) was available during the training stage.

### Classical multidimensional scaling (MDS)

Classical multidimensional scaling takes a pairwise distance matrix (*n*×*n* matrix, for *n* input items) as input, and produces *n* points in a *q*-dimensional Euclidean space, where *q*≤*n*−1. More specifically, the output is an *n*×*q* coordinate matrix, where each row corresponds to one of the *n* input items, and that row contains the *q* coordinates of the corresponding item-representing point [11]. The Euclidean distance between each pair of points is meant to approximate the distance between the corresponding two items in the original distance matrix.

These points can then be simultaneously visualized in a 2- or 3-dimensional space by taking the first 2, respectively 3, coordinates (out of *q*) of the coordinate matrix. The result is a Molecular Distance Map [46], and the MoDMap of a genomic dataset represents a visualization of the simultaneous interrelationships among all DNA sequences in the dataset.

### Software implementation

The algorithms for ML-DSP were implemented using the software package MATLAB R2017A, license no. 964054, as well as the open-source toolbox Fathom Toolbox for MATLAB [58] for distance computation. All software can be downloaded from https://github.com/grandhawa/MLDSP. The user can use this code to reproduce all results in this paper, and also has the option to input their own dataset and use it as training set for the purpose of classifying new genomic DNA sequences.

All experiments were performed on an ASUS ROG G752VS computer with 4 cores (8 threads) of a 2.7GHz Intel Core i7 6820HK processor and 64GB DD4 2400MHz SDRAM.

### Datasets

All datasets in this paper can be found at https://github.com/grandhawa/MLDSP in the “DataBase” directory. The mitochondrial dataset comprises all of the 7396 complete reference mtDNA sequences available in the NCBI Reference Sequence Database RefSeq on June 17, 2017. We performed computational experiments on several different subsets of this dataset. The bacteria dataset comprises all 4710 complete bacterial genomes with lengths between 20,000 bp and 500,000 bp, available in the aforementioned NCBI database on the same date. The dengue virus dataset contained all 4721 dengue virus genomes available in the NCBI database on August 10,2017. Note that any letters “N” in these DNA sequences were deleted.

For the performance comparison between ML-DSP and other alignment-free and alignment-based methods we also used the benchmark datasets of 38 influenza virus sequences, and 41 mammalian complete mtDNA sequences from [47].

## Results and discussion

Following the design and implementation of the ML-DSP genomic sequence classification tool prototype, we investigated which type of length-normalization and which type of distance were most suitable for genome classification using this method. We then conducted a comprehensive analysis of the various numerical representations of DNA sequences used in the literature, and determined the top three performers. Having set the main parameters (length-normalization method, distance, and numerical representation), we tested ML-DSP’s ability to classify mtDNA genomes at taxonomic levels ranging from the domain level down to the genus level, and obtained average levels of classification accuracy of >97*%*. Finally, we compared ML-DSP with other alignment-based and alignment-free genome classification methods, and showed that ML-DSP achieved higher accuracy and significantly higher speeds.

### Analysis of distances and of length normalization approaches

To decide which distance measure and which length normalization method were most suitable for genome comparisons with ML-DSP, we used nine different subsets of full mtDNA sequences from our dataset. These subsets were selected to include most of the available complete mtDNA genomes (Vertebrates dataset of 4322 mtDNA sequences), as well as subsets containing similar sequences, of similar length (Primates dataset of 148 mtDNA sequences), and subsets containing mtDNA genomes showing large differences in length (Plants dataset of 174 mtDNA sequences).

The classification accuracy scores obtained using the two considered distance measures (Euclidean and Pearson Correlation Coefficient) and two different length-normalization approaches (normalization to maximum length and normalization to median length) on several datasets are listed in Table 2. The classification accuracy scores are slightly higher for PCC, but sufficiently close to those obtained when using the Euclidean distance to be inconclusive.

**Table 2:** Maximum classification accuracy scores when using Euclidean vs. Pearson’s correlation coefficient (PCC) as a distance measure

					Maximum accuracy			
					Euclidean		PCC	
Data Set	No. of Seq.	Max Length (bp)	Min Length (bp)	Median Length (bp)	Norm. to Max Length (a)	Norm. to Median Length (b)	Norm. to Max Length (c)	Norm. to Median Length (d)
Primates (Haplorrhini: 115, Strepsirrhini: 33)	148	17531	15467	16554	98.6%	100%	100%	100%
Protists (Alveolata: 34, Rhodophyta: 46, Stramenopiles: 79)	159	77356	5882	35660	89.3%	90.6%	96.2%	91.2%
Fungi (Basidiomycota: 30, Pezizomycotina: 104, Saccharomycotina:92)	226	235849	1364	39154	70.1%	82.6%	87.9%	89.3%
Plants (Chlorophyta: 44, Streptophyta: 130)	174	1999595	12998	128211	95.4%	94.8%	90.2%	91.4%
Amphibians (Anura: 161, Caudata:95, Gymnophiona: 34)	290	28757	15757	17271	95.2%	97.6%	98.3%	99.0%
Mammals (Xenarthrans: 30, Bats: 54, Carnivores: 135, Even-toed Ungulates: 242, Insectivores: 40, Marsupials: 34, Primates: 148, Rodents and Rabbits: 147)	830	17734	15289	16537	95.2%	96.1%	97.8%	97.1%
Insects (Coleoptera: 95, Dictyptera: 77, Diptera: 149, Hemiptera: 126, Hymenoptera: 47, Lepidoptera:294, Orthoptera: 110)	898	20731	10662	15529	87.9%	90.0%	91.3%	94.2%
3 classes (Amphibians: 290, Mammals: 874, Insects: 1006)	2170	28757	8118	16361	99.9%	99.7%	99.8%	99.7%
Vertebrates (Amphibians: 290, Birds: 553, Fish: 2313, Mammals: 874, Reptiles: 292)	4322	28757	14935	16616	99.6%	99.8%	99.6%	99.7%
**Table Average Accuracy**	——	——	——	——	92.4%	94.6%	95.7%	95.7%

(a)(c) Genomes normalized to the maximum genome sequence length; (b)(d) Genomes normalized to the median genome sequence length

In the remainder of this paper we chose the Pearson Correlation Coefficient because it is scale independent (unlike the Euclidean distance, which is, e.g., sensitive to the offset of the signal, whereby signals with the same shape but different starting points are regarded as dissimilar [59]), and the length-normalization to median length because it is economic in terms of memory usage.

### Analysis of various numerical representations of DNA sequences

We analyzed the effect on the ML-DSP classification accuracy of thirteen different one-dimensional numeric representations for DNA sequences, grouped as: Fixed mappings DNA numerical representations (Table 1 representations #1, #2, #3, #6, #7, see [29], and representations #10, #11, #12, #13 - which are one-dimensional variants of the binary representation proposed in [29]), mappings based on some physio-chemical properties of nucleotides (Table 1 representation #4, see [29, 32], and representation #5, see [29, 31, 32]), and mappings based on the nearest-neighbour values (Table 2 representations #8, #9, see [30]).

The datasets used for this analysis were the same as those in Table 2. The supervised machine learning classifiers used for this analysis were the six classifiers listed in the Methods and Implementation section, with the exception of the datasets with more than 2000 sequences where two of the classifiers (Subspace Discriminant and Subspace KNN) were omitted as being too slow. The results and the average accuracy scores for all these numerical representations, classifiers and datasets are summarized in Table 3.

**Table 3:** Average classification accuracies for 13 numerical representations. Averages over the six classifiers are in bold

DataSet/	Numerical representation												
classification model	Integer	Integer (Other)	Real	Atomic	EIIP	PP	Paired Num.	NN based doublet	Codon	Just-A	Just-C	Just-G	Just-T
Primates (148 sequences)													
Linear Discriminant	97.3%	98.0%	99.3%	98.6%	99.3%	99.3%	97.3%	97.3%	98.0%	98.0%	97.3%	96.6%	96.6%
Linear SVM	97.3%	95.9%	98.6%	96.6%	97.3%	98.0%	95.9%	97.3%	94.6%	98.0%	96.6%	96.6%	95.3%
Quadratic SVM	97.3%	95.9%	98.6%	93.2%	95.9%	98.6%	96.6%	98.6%	95.9%	98.0%	98.0%	97.3%	95.9%
Fine KNN	98.0%	98.0%	100.0%	98.0%	96.6%	100.0%	99.3%	99.3%	98.0%	100.0%	98.6%	100.0%	98.6%
Subspace Discriminant	98.0%	97.3%	99.3%	98.0%	99.3%	98.6%	95.3%	97.3%	95.9%	98.0%	97.3%	98.0%	95.3%
Subspace KNN	98.0%	97.3%	98.6%	96.6%	95.9%	98.0%	100%	98.0%	98.0%	99.3%	97.3%	98.6%	98.6%
Average	**97.7%**	**97.1%**	**99.1%**	**96.8%**	**97.4%**	**98.8%**	**97.4%**	**98.0%**	**96.7%**	**98.6%**	**97.5%**	**97.9%**	**96.7%**
Protists (159 sequences)													
Linear Discriminant	83.6%	84.9%	85.5%	86.2%	86.2%	84.3%	85.5%	83.0%	85.5%	84.3%	83.6%	83.0%	83.6%
Linear SVM	84.3%	83.0%	83.6%	83.0%	83.0%	71.7%	82.4%	83.0%	83.6%	83.6%	83.6%	83.6%	83.0%
Quadratic SVM	84.9%	84.9%	83.6%	82.4%	83.0%	81.1%	85.5%	84.9%	86.2%	83.0%	84.3%	83.0%	86.2%
Fine KNN	86.8%	86.2%	81.8%	84.3%	88.1%	78.0%	89.9%	88.7%	91.8%	86.8%	88.7%	93.7%	92.5%
Subspace Discriminant	85.5%	84.9%	88.1%	86.8%	85.5%	86.8%	83.6%	83.0%	85.5%	84.9%	83.6%	83.0%	83.6%
Subspace KNN	88.7%	87.4%	91.8%	85.5%	88.1%	91.2%	89.9%	88.1%	93.1%	86.8%	88.1%	92.5%	93.7%
Average	**85.6%**	**85.2%**	**85.7%**	**84.7%**	**85.7%**	**82.2%**	**86.1%**	**85.1%**	**87.6%**	**84.9%**	**85.3%**	**86.5%**	**87.1%**
Fungi (226 sequences)													
Linear Discriminant	76.3%	76.8%	82.1%	50.9%	57.1%	80.4%	75.4%	68.8%	77.7%	81.7%	70.5%	71.9%	79.0%
Linear SVM	66.5%	58.0%	76.8%	49.1%	46.0%	73.7%	73.2%	66.1%	71.0%	75.9%	64.7%	66.1%	75.4%
Quadratic SVM	58.9%	59.8%	82.6%	33.9%	37.9%	79.9%	71.4%	67.4%	63.4%	71.0%	67.9%	71.4%	64.3%
Fine KNN	61.6%	56.7%	84.4%	49.6%	54.9%	85.7%	72.3%	65.2%	58.0%	68.8%	61.6%	68.8%	67.9%
Subspace Discriminant	74.6%	75.0%	78.6%	46.0%	55.4%	79.0%	75.0%	71.4%	78.1%	79.9%	68.8%	69.2%	78.6%
Subspace KNN	63.4%	58.9%	89.3%	51.8%	58.0%	89.3%	68.3%	63.8%	59.8%	67.9%	65.6%	72.8%	64.3%
Average	**66.9%**	**64.2%**	**82.3%**	**46.9%**	**51.6%**	**81.3%**	**72.6%**	**67.1%**	**68.0%**	**74.2%**	**66.5%**	**70.0%**	**71.6%**
Plants (174 sequences)													
Linear Discriminant	96.0%	95.4%	76.4%	92.5%	93.7%	91.4%	95.4%	96.0%	95.4%	96.0%	96.0%	96.0%	96.0%
Linear SVM	96.0%	96.0%	85.6%	96.0%	96.0%	87.9%	94.8%	96.0%	96.0%	96.0%	96.0%	96.0%	96.0%
Quadratic SVM	96.0%	96.0%	86.8%	96.0%	96.0%	88.5%	94.3%	96.0%	96.0%	96.0%	96.0%	96.0%	96.0%
Fine KNN	93.1%	94.8%	91.4%	94.3%	94.3%	90.8%	86.8%	93.1%	94.3%	93.7%	91.4%	93.1%	93.1%
Subspace Discriminant	96.0%	95.4%	87.4%	94.8%	95.4%	87.9%	94.8%	96.0%	96.0%	96.0%	96.0%	96.0%	96.0%
Subspace KNN	93.7%	94.3%	90.2%	94.3%	94.3%	90.2%	92.5%	92.5%	94.8%	93.7%	94.3%	94.8%	94.3%
Average	**95.1%**	**95.3%**	**86.3%**	**94.7%**	**95.0%**	**89.5%**	**93.1%**	**94.9%**	**95.4%**	**95.2%**	**95.0%**	**95.3%**	**95.2%**
Amphibians (290 sequences)													
Linear Discriminant	92.1%	91.4%	95.5%	89.0%	89.3%	99.0%	94.5%	93.4%	91.4%	96.2%	93.4%	93.8%	91.7%
Linear SVM	91.0%	90.0%	89.0%	88.3%	88.6%	93.1%	89.0%	91.4%	90.0%	93.1%	92.1%	92.4%	90.3%
Quadratic SVM	90.3%	89.0%	92.4%	59.3%	83.4%	96.6%	91.0%	93.1%	86.9%	94.1%	93.1%	93.4%	90.7%
Fine KNN	90.0%	86.9%	96.6%	83.8%	83.4%	98.3%	87.9%	92.1%	89.7%	93.4%	91.7%	94.8%	89.7%
Subspace Discriminant	90.7%	90.3%	90.0%	89.3%	89.3%	96.6%	90.3%	91.7%	90.3%	95.2%	92.8%	92.1%	91.0%
Subspace KNN	88.3%	86.6%	94.1%	85.2%	84.5%	98.3%	89.7%	92.8%	87.2%	94.5%	90.0%	94.8%	90.3%
Average	**90.4%**	**89.0%**	**92.9%**	**82.5%**	**86.4%**	**97.0%**	**90.4%**	**92.4%**	**89.3%**	**94.4%**	**92.2%**	**93.6%**	**90.6%**
Mammals (830 sequences)													
Linear Discriminant	98.3%	97.6%	97.7%	97.0%	96.0%	97.1%	96.6%	97.2%	96.7%	98.0%	96.9%	96.3%	96.3%
Linear SVM	90.6%	89.6%	88.9%	84.5%	85.3%	91.6%	86.5%	91.2%	88.8%	90.8%	90.0%	88.2%	88.1%
Quadratic SVM	92.4%	89.9%	91.0%	32.9%	41.7%	93.4%	88.0%	93.4%	89.9%	90.7%	92.5%	89.8%	90.5%
Fine KNN	94.1%	92.3%	96.0%	79.9%	81.0%	96.6%	93.9%	93.7%	91.7%	96.3%	96.3%	94.8%	95.5%
Subspace Discriminant	92.3%	91.9%	92.3%	88.3%	87.7%	94.0%	90.2%	91.7%	90.4%	92.3%	93.4%	91.9%	91.3%
Subspace KNN	92.8%	90.8%	95.5%	78.2%	79.2%	96.4%	91.2%	93.3%	89.2%	94.8%	94.3%	94.9%	92.2%
Average	**93.4%**	**92.0%**	**93.6%**	**76.8%**	**78.5%**	**94.9%**	**91.1%**	**93.4%**	**91.1%**	**93.8%**	**93.9%**	**92.7%**	**92.3%**
Insects (898 sequences)													
Linear Discriminant	92.2%	92.7%	90.1%	91.6%	92.2%	94.2%	93.3%	92.4%	89.2%	93.1%	92.1%	94.4%	90.4%
Linear SVM	86.9%	82.6%	85.9%	66.7%	69.5%	85.3%	86.4%	90.0%	80.5%	89.4%	87.4%	88.4%	86.2%
Quadratic SVM	85.0%	81.8%	86.7%	24.4%	21.3%	87.1%	85.7%	89.6%	82.6%	89.5%	88.0%	89.6%	85.3%
Fine KNN	82.0%	79.3%	80.0%	62.5%	68.0%	93.2%	83.3%	87.9%	80.8%	85.6%	83.6%	87.9%	83.0%
Subspace Discriminant	85.7%	83.9%	88.3%	77.5%	79.3%	89.1%	88.0%	88.2%	82.1%	87.1%	87.6%	88.2%	86.4%
Subspace KNN	80.4%	77.3%	90.5%	61.0%	67.6%	92.0%	81.4%	86.9%	77.4%	85.4%	86.0%	89.3%	81.4%
Average	**85.4%**	**82.9%**	**86.9%**	**64.0%**	**66.3%**	**90.2%**	**86.4%**	**89.2%**	**82.1%**	**88.4%**	**87.5%**	**89.6%**	**85.5%**
3Classes (2170 sequences; Subspace Discriminant & Subspace KNN omitted)													
Linear Discriminant	99.9%	99.9%	99.6%	99.4%	99.7%	99.7%	99.7%	99.7%	99.8%	99.8%	99.9%	99.9%	99.6%
Linear SVM	94.1%	90.2%	99.4%	89.8%	89.3%	99.6%	99.2%	98.1%	94.6%	99.1%	97.3%	99.3%	97.9%
Quadratic SVM	97.5%	92.5%	99.4%	66.6%	78.8%	99.7%	99.5%	98.7%	97.6%	99.4%	98.4%	99.5%	98.8%
Fine KNN	95.9%	95.2%	97.6%	93.3%	94.4%	95.9%	97.6%	97.7%	96.4%	98.9%	98.0%	99.2%	98.4%
Average	**96.9%**	**94.5%**	**99.0%**	**87.3%**	**90.6%**	**98.7%**	**99.0%**	**98.6%**	**97.1%**	**99.3%**	**98.4%**	**99.5%**	**98.7%**
Vertebrates (4322 sequences; Subspace Discriminant & Subspace KNN omitted)													
Linear Discriminant	99.7%	99.7%	99.6%	99.3%	99.5%	99.7%	99.2%	99.3%	99.3%	99.3%	99.4%	99.5%	99.2%
Linear SVM	98.3%	98.2%	98.5%	96.3%	96.8%	97.9%	98.0%	98.4%	98.2%	98.2%	98.5%	98.8%	98.4%
Quadratic SVM	98.1%	96.6%	99.0%	40.6%	34.0%	98.7%	98.4%	98.2%	96.7%	98.5%	98.7%	98.8%	98.6%
Fine KNN	97.1%	96.1%	98.4%	88.3%	91.7%	97.9%	96.4%	96.3%	95.3%	96.4%	97.5%	97.6%	97.2%
Average	**98.3%**	**97.7%**	**98.9%**	**81.1%**	**80.5%**	**98.6%**	**98.0%**	**98.1%**	**97.4%**	**98.1%**	**98.5%**	**98.7%**	**98.4%**
**Table average**	**90.0%**	**88.7%**	**91.6%**	**79.4%**	**81.3%**	**92.3%**	**90.5%**	**90.7%**	**89.4%**	**91.9%**	**90.5%**	**91.5%**	**90.7%**

As can be observed from Table 3, for all numerical representations, the table average accuracy scores (last row: average of averages, first over the six classifiers for each dataset, and then over all datasets), are high. Surprisingly, even using a single nucleotide numerical representation, which treats three of the nucleotides as being the same, and singles out only one of them (“Just-A”), results in an average accuracy of 91.9%. The best accuracy, for these datasets, is achieved when using the “PP” representation, which yields an average accuracy of 92.3%.

For subsequent experiments we selected the top three representations in terms of accuracy scores: “PP”, “Just-A”, and “Real” numerical representations.

### ML-DSP for three classes of vertebrates

**Fig. 3:**
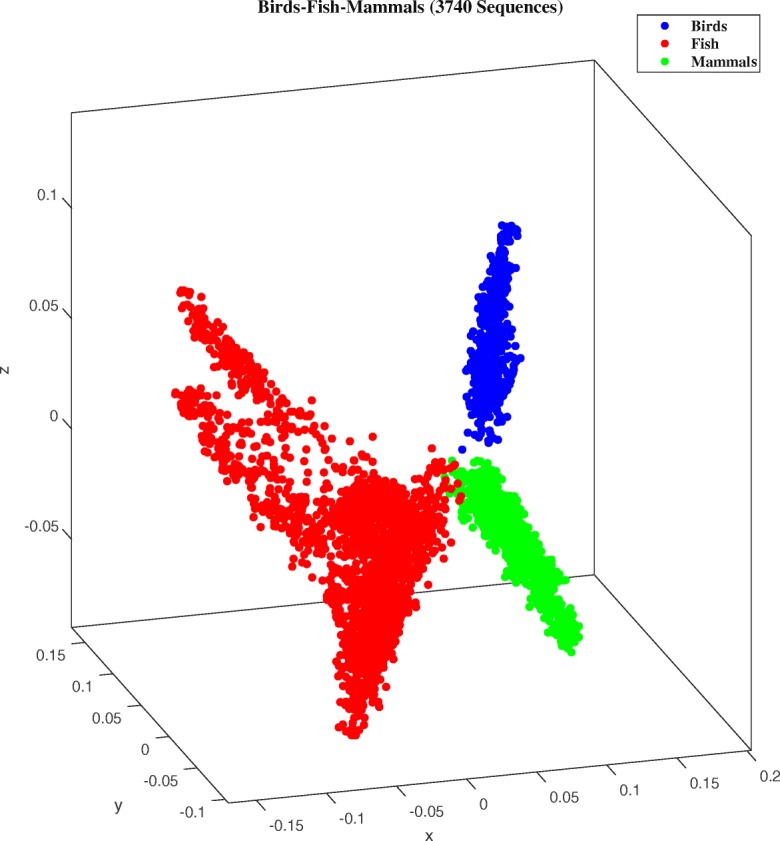
MoDMap of 3740 full mtDNA genomes in subphylum Vertebrata, into three classes: Birds (blue, Aves: 553 genomes), fish (red, Actinopterygii 2176 genomes, Chondrichthyes 130 genomes, Coelacanthiformes 2 genomes, Dipnoi 5 genomes), and mammals (green, Mammalia: 874 genomes). The accuracy of the ML-DSP classification into three classes, using the Quadratic SVM classifier, with the “PP” numerical representation, and PCC between magnitude spectra of DFT, was 100%

As an application of ML-DSP using the “PP” numerical representation for DNA sequences, we analyzed the set of vertebrate mtDNA genomes (median length 16,606 bp). The MoDMap, i.e., the multi-dimensional scaling 3D visualization of the genome interrelationships as described by the distances in the distance matrix, is illustrated in Fig. 3. The dataset contains 3740 complete mtDNA genomes: 553 bird genomes, 2313 fish genomes, and 874 mammalian genomes. Quantitatively, the classification accuracy score obtained by the Quadratic SVM classifier was 100%.

### Classifying genomes with ML-DSP, at all taxonomic levels

We tested the ability of ML-DSP to classify complete mtDNA sequences at various taxonomic levels. For every dataset, we tested using the “PP”, “Just-A”, and “Real” numerical representations.

The starting point was domain Eukaryota (7396 sequences), which was classified into kingdoms, then kingdom Animalia was classified into phyla, etc. At each level, we picked the cluster with the highest number of sequences and then classified it into the next taxonomic level sub-clusters. The lowest level classified was family Cyprinidae (81 sequences) into its six genera. For each dataset, we tested all six classifiers, and the maximum of these six classification accuracy scores for each dataset are shown in Table 4.

**Table 4:** Maximum classification accuracy (of the accuracies obtained with each of the six classifiers) of ML-DSP, for datasets at different taxonomic levels, from ‘domain into kindgoms’ down to ‘family into genera’

Test	No. of	Max	Min	Median	Mean	Numerical representation maximum accuracy				
	Seq.	Length	Length	Length	Length	PP	Real	Just-A	Random3^*^	Random13^**^
Domain to Kingdom	7396	1999595	1136	16580	25434	96.2%	97.3%	96.1%	95.5%	92.8%
Domain:Eukaryota										
Kingdoms:										
Plants:,254, Animals: 6697,										
Fungi: 267, Protists :178										
Domain to Kingdom (No Protists)	7218	1999595	1136	16573	25254	97.9%	98.4%	97.9%	97.4%	94.4%
Domain:Eukaryota										
Kingdoms:										
Plants:254, Animals: 6697,										
Fungi: 267										
Kingdom to Phylum	6673	48161	5596	16553	16474	96.2%	95.9%	95.3%	93.6%	85.6%
Kingdom: Animalia										
Phylum:										
Chordata:4367, Cnidaria: 127,										
Ecdysozoa: 1572, Porifera: 60,										
Echinodermata: 44, Lophotrochozoa: 403,										
Platyhelminthes: 100										
Phylum to SubPhylum	4367	28757	13424	16615	16791	99.7%	99.8%	99.8%	99.5%	99.7%
Phylum:Chordata										
SubPhylum:Cephalochordata:9,										
Craniata: 4334, Tunicata:24										
SubPhylum to Class	4322	28757	14935	16616	16806	99.7%	99.6%	99.3%	99.2%	86.2%
SubPhylum:Vertebrata										
Class:										
Amphibians(Amphibia):290,										
Birds(Aves): 553,										
Fish(Actinopterygii, Chondrichthyes,										
Dipnoi, Coelacanthiformes): 2313,										
Mammals(Mammalia): 874,										
Reptiles(Crocodylia, Sphenodontia,										
Squamata, Testudines): 292										
Class to SubClass	2176	22217	15534	16589	16656	100%	99.9%	99.9%	99.8%	99.2%
Class:Actinopterygii										
SubClass:										
Chondrostei: 24, Cladistia: 11,										
Neopterygii: 2141										
SubClass to SuperOrder	1488	22217	15534	16597	16669	96.2%	96.4%	95.4%	94.4%	78.8%
SubClass: Neopterygii										
SuperOrder:										
Osteoglossomorpha:23, Elopomorpha: 60,										
Clupeomorpha: 75, Ostariophysi: 792,										
Protacanthopterygii: 66, Paracanthopterygii: 46,										
Acanthopterygii:426										
SuperOrder to Order	781	17859	16123	16597	16621	99.0%	98.7%	98.8%	97.6%	92.2%
SuperOrder:Ostariophysi										
Order:										
Cypriniformes: 643, Characiformes: 31,										
Siluriformes: 107										
Order to family	635	17859	16411	16601	16627	98.9%	97.8%	98.3%	97.3%	85.7%
Order: Cypriniformes										
Family:										
Balitoridae: 25, Catostomidae:12,										
Cobitidae: 51, Cyprinidae: 502,										
Nemacheilidae: 47										
Family to Genus	81	17155	16563	16597	16630	91.8%	92.6%	91.4%	85.2%	66.7%
Family: Cyprinidae										
Genus:										
*Schizothorax*: 19, *Labeo*: 19,										
*Acrossocheilus*: 12, *Acheilognathus*: 10,										
*Rhodeus*: 11, *Onychostoma*: 10										
Table Average Accuracy	—–	—–	—–	—–	—–	97.6%	97.6%	97.2%	96.0%	88.1%

At each level, the cluster with the highest number of sequences was chosen as the next dataset to be classified into its sub-taxa. *Random3: each sequence is represented by a random representation among PP, Real, or Just-A. **Random13: each sequence is represented by random representation among 13 representations (Integer, Integer(Other), Real, Atomic, EIIP, PP, Paired Numeric, Nearest neighbor based doublet, Codon, Just-A, Just-C, Just-G or Just-T)

Note that, at each taxonomic level, the maximum classification accuracy scores (among the six classifiers) for each of the three numerical representations considered are high, ranging from 91.4% to 100%, with only three scores under 95%. As this analysis also did not reveal a clear winner among the top three numerical representations, the question then arose whether the numerical representation we use mattered at all. To answer this question, we performed two additional experiments, that exploit the fact that the Pearson correlation coefficient is scale independent, and only looks for a pattern while comparing signals. For the first experiment we selected the top three numerical representations (“PP”, “Just-A”, and “Real”) and, for each sequence in a given dataset, a numerical representation among these three was randomly chosen, with equal probability, to be the digital signal that represents it. The results are shown under the column “Random3” in Table 4: The maximum accuracy score over all the datasets is 96%. This is almost the same as the accuracy obtained when one particular numerical representation was used (1% lower, which is well within experimental error). We then repeated this experiment, this time picking randomly from any of the thirteen numerical representations considered. The results are shown under the column “Random13” in Table 4, with the table average accuracy score being 88.1%.

Overall, our results suggest that all three numerical representations “PP”, “Just-A”, and “Real” have very high classifications accuracy scores (average >97%), and even a random choice of one of these representations for each sequence in the dataset does not significantly affect the classification accuracy score of ML-DSP (average 96%).

We also note that, in addition to being highly accurate in its classifications, ML-DSP is ultrafast. Indeed, even for the largest dataset in Table 2, subphylum Vertebrata (4322 complete mtDNA genomes, average length 16,806 bp), the distance matrix computation (which is the bulk of the classification computation) lasted under 5 s. Classifying a new primate mtDNA genome took 0.06 s when trained on 148 primate mtDNA genomes, and classifying a new vertebrate mtDNA genome took 7 s when trained on the 4322 vertebrate mtDNA genomes. The result was updated with an experiment whereby QSVM was trained on the 4322 complete vertebrate genomes in Table 2, and querried on the 694 new vertebrate mtDNA genomes uploaded on NCBI between June 17, 2017 and January 7, 2019. The accuracy of classification was 99.6%, with only three reptile mtDNA genomes mis-classified as amphibian genomes: *Bavayia robusta*, robust forest bavayia - a species of gecko, NC_034780, *Mesoclemmys hogei*, Hoge’s toadhead turtle, NC_036346, and *Gonatodes albogularis*, yellow-headed gecko, NC_035153.

### MoDMap visualization vs. ML-DSP quantitative classification results

The hypothesis tested by the next experiments was that the quantitative accuracy of the classification of DNA sequences by ML-DSP would be significantly higher than suggested by the visual clustering of taxa in the MoDMap produced with the same pairwise distance matrix.

**Fig. 4:**
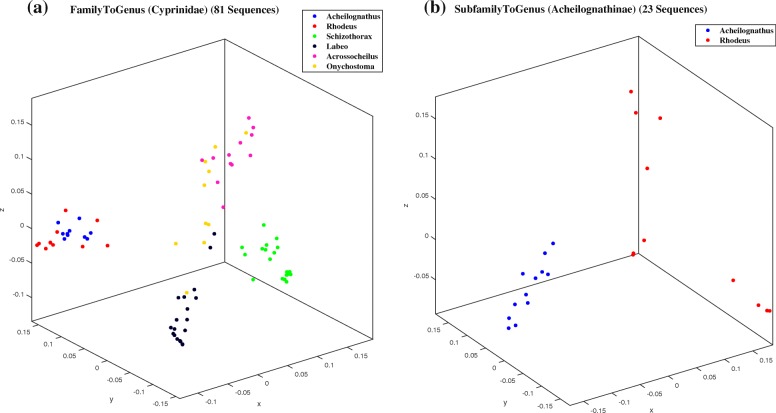
MoDMap of family Cyprinidae and its genera. (**a**): Genera *Acheilognathus* (blue, 10 genomes), *Rhodeus* (red, 11 genomes), *Schizothorax* (green, 19 genomes), *Labeo* (black, 19 genomes), *Acrossocheilus* (magenta, 12 genomes), *Onychostoma* (yellow, 10 genomes); (**b**): Genera *Acheilognathus* and *Rhodeus*, which overlapped in (**a**), are visually separated when plotted separately in (**b**). The classification accuracy with Quadratic SVM of the dataset in (**a**) was 91.8%, and of the dataset in (**b**) was 100%

As an example, the MoDMap in Fig. 4a, visualizes the distance matrix of mtDNA genomes from family Cyprinidae (81 genomes) with its genera *Acheilognathus* (10 genomes), *Rhodeus* (11 genomes), *Schizothorax* (19 genomes), *Labeo* (19 genomes), *Acrossocheilus* (12 genomes), *Onychostoma* (10 genomes); only the genera with at least 10 genomes are considered. The MoDMap seems to indicate an overlap between the clusters *Acheilognathus* and *Rhodeus*, which is biologically plausible as these genera belong to the same sub-family Acheilognathinae. However, when zooming in by plotting a MoDMap of only these two genera, as shown in Fig. 4b, one can see that the clusters are clearly separated visually. This separation is confirmed by the fact that the accuracy score of the Quadratic SVM classifier for the dataset in Fig. 4b is 100%. The same quantitative accuracy score for the classification of the dataset in Fig. 4a with Quadratic SVM is 91.8%, which intuitively is much better than the corresponding MoDMap would suggest. This is likely due to the fact that the MoDMap is a three-dimensional approximation of the positions of the genome-representing points in a multi-dimensional space (the number of dimensions is (*n*−1), where *n* is the number of sequences).

This being said, MoDMaps can still serve for exploratory purposes. For example, the MoDMap in Fig. 4a suggests that species of the genus *Onychostoma* (subfamily listed “unknown” in NCBI) (yellow), may be genetically related to species of the genus *Acrossocheilus* (subfamily Barbinae) (magenta). Upon further exploration of the distance matrix, one finds that indeed the distance between the centroids of these two clusters is lower than the distance between each of these two cluster-centroids to the other cluster-centroids. This supports the hypotheses, based on morphological evidence [60], that genus *Onychostoma* belongs to the subfamily Barbinae, respectively that genus *Onychostoma* and genus *Acrossocheilus* are closely related [61]. Note that this exploration, suggested by MoDMap and confirmed by calculations based on the distance matrix, could not have been initiated based on ML-DSP alone (or other supervised machine learning algorithms), as ML-DSP only predicts the classification of new genomes into one of the taxa that it was trained on, and does not provide any other additional information.

**Fig. 5:**
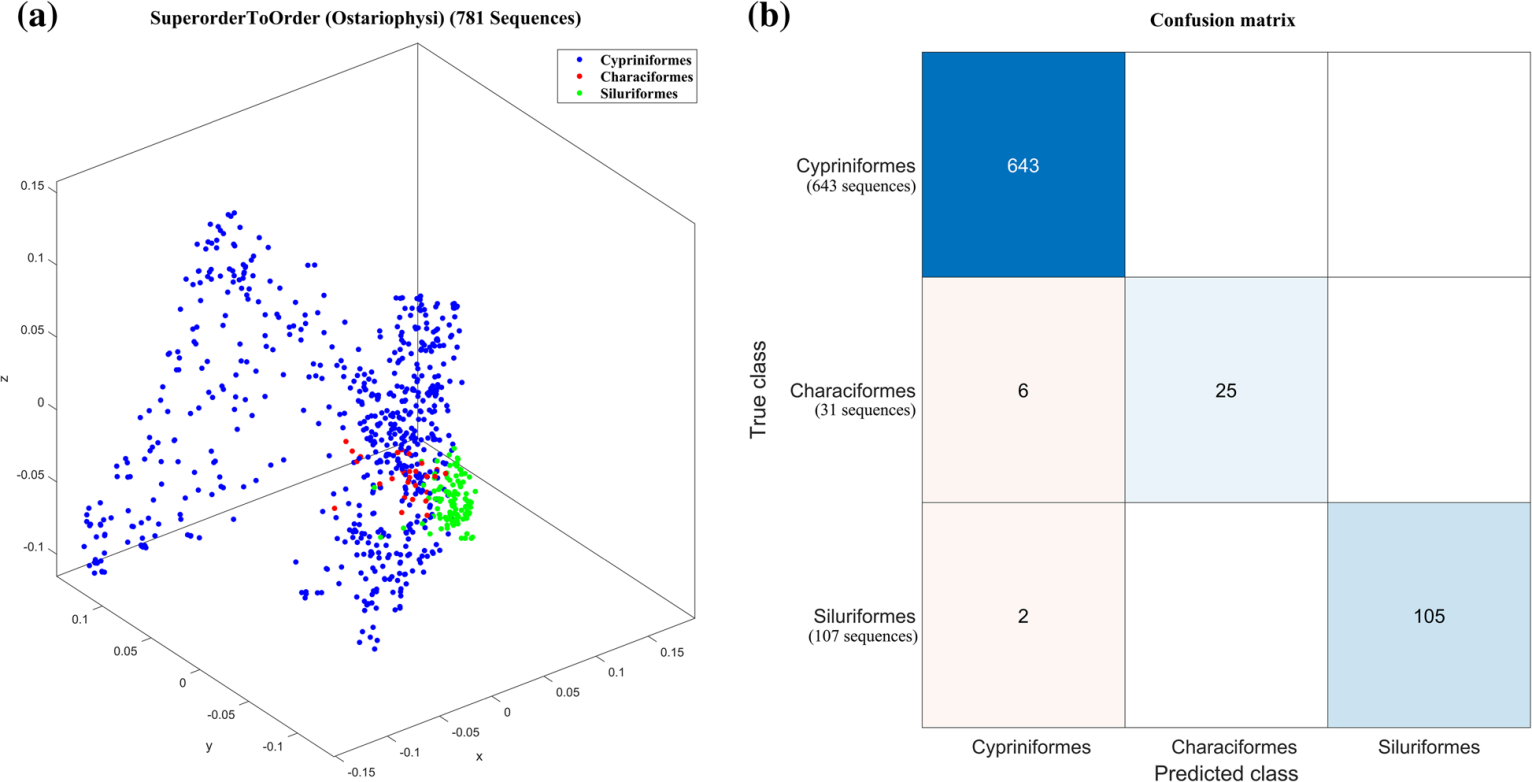
MoDMap of the superorder Ostariophysi, and the confusion matrix for the Quadratic SVM classification of this superorder into orders. (**a**): MoDMap of orders Cypriniformes (blue, 643 genomes), Characiformes (red, 31 genomes), Siluriformes (green, 107 genomes). (**b**): The confusion matrix generated by Quadratic SVM, illustrating its true class vs. predicted class performance (top-to-bottom and left-to-right: Cypriniformes, Characiformes, Siluriformes). The numbers in the squares on the top-left to bottom-right diagonal (blue) indicate the numbers of correctly classified DNA sequences, by order. The off-diagonal pink squares indicate that 6 mtDNA genomes of the order Characiformes have been erroneously predicted to belong to the order Cypriniformes (center-left), and 2 mtDNA genomes of the order Siluriformes have been erroneously predicted to belong to the order Cypriniformes (bottom-left). The Quadratic SVM that generated this confusion matrix had a 99% classification accuracy

As another comparison point between MoDMaps and supervised machine learning outputs, Fig. 5a shows the MoDMap of the superorder Ostariophysi with its orders Cypriniformes (643 genomes), Characiformes (31 genomes) and Siluriformes (107 genomes). The MoDMap shows the clusters as overlapping, but the Quadratic SVM classifier that quantitatively classifies these genomes has an accuracy of 99%. Indeed, the confusion matrix in Fig. 5b shows that Quadratic SVM mis-classifies only 8 sequences out of 781 (recall that, for *m* clusters, the *m*×*m* confusion matrix has its rows labelled by the true classes and columns labelled by the predicted classes; the cell (*i*,*j*) shows the number of sequences that belong to the true class *i*, and have been predicted to be of class *j*). This indicates that when the visual representation in a MoDMap shows cluster overlaps, this may only be due to the dimensionality reduction to three dimensions, while ML-DSP actually provides a much better quantitative classification based on the same distance matrix.

### Applications to other genomic datasets

**Fig. 6:**
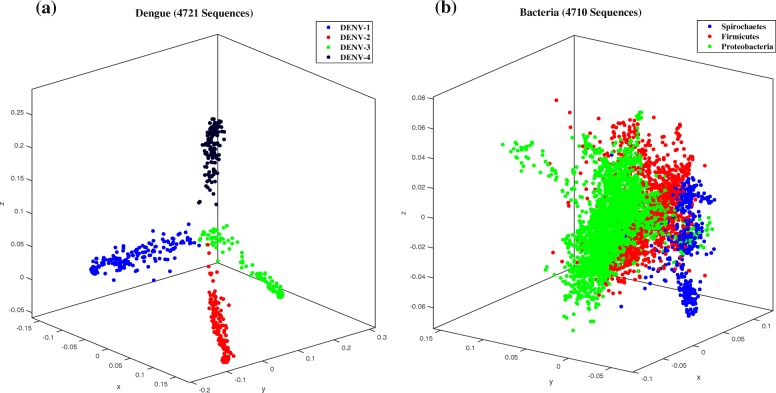
(**a**) MoDMap of 4271 dengue virus genomes. The colours represent virus subtypes DENV-1 (blue, 2008 genomes), DENV-2 (red, 1349 genomes), DENV-3 (green, 1010 genomes), DENV-4 (black, 354 genomes); The classification accuracy of the Quadratic SVM classifier for this dataset was 100%. (**b**) MoDMap of 4710 bacterial genomes. The colours represent bacterial phyla: Spirochaetes (blue, 437 genomes), Firmicutes (red, 1129 genomes), Proteobacteria (green, 3144 genomes). The accuracy of the Quadratic SVM classifier for this dataset was 95.5%

The two experiments in this section indicate that the applicability of our method is not limited to mitochondrial DNA sequences. The first experiment, Fig. 6a, shows the MoDMap of all 4721 complete dengue virus sequences available in NCBI on August 10,2017, classified into the subtypes DENV-1 (2008 genomes), DENV-2 (1349 genomes), DENV-3 (1010 genomes), DENV-4 (354 genomes). The average length of these complete viral genomes is 10,595 bp. Despite the dengue viral genomes being very similar, the classification accuracy of this dataset into subtypes, using the Quadratic SVM classifier, was 100%. The second experiment, Fig. 6b, shows the MoDMap of 4710 bacterial genomes, classified into three phyla: Spirochaetes (437 genomes), Firmicutes (1129 genomes), and Proteobacteria (3144 genomes). The average length of these complete bacterial genomes is 104,150 bp, with the maximum length being 499,136 bp and the minimum length being 20,019 bp. The classification accuracy of the Quadratic SVM classifier for this dataset was 95.5%.

### Comparison of ML-DSP with state-of-the-art alignment-based and alignment-free tools

The computational experiments in this section compare ML-DSP with three state-of-the-art alignment-based and alignment-free methods: the alignment-based tool MEGA7 [3] with alignment using MUSCLE [4] and CLUSTALW [5, 6], and the alignment-free method FFP (Feature Frequency Profiles) [28].

For this performance analysis we selected three datasets. The first two datasets are benchmark datasets used in other genetic sequence comparison studies [47]: The first dataset comprises 38 influenza viral genomes, and the second dataset comprises 41 mammalian complete mtDNA sequences. The third dataset, of our choice, is much larger, consisting of 4,322 vertebrate complete mtDNA sequences, and was selected to compare scalability.

For the alignment-based methods, we used the distance matrix calculated in MEGA7 from sequences aligned with either MUSCLE or CLUSTALW. For the alignment-free FFP, we used the default value of *k*=5 for *k*-mers (a *k*-mer is any DNA sequence of length *k*; any increase in the value of the parameter *k*, for the first dataset, resulted in a lower classification accuracy score for FFP). For ML-DSP we chose the Integer numerical representation and computed the average classification accuracy over all six classifiers for the first two datasets, and over all classifiers except Subspace Discriminant and Subspace KNN for the third dataset.

Table 5 shows the performance comparison (classification accuracy and processing time) of these four methods. The processing time included all computations, starting from reading the datasets to the completion of the distance matrix - the common element of all four methods. The listed processing times do not include the time needed for the computation of phylogenetic trees, MoDMap visualizations, or classification.

**Table 5:** Comparison of classification accuracy and processing time for the distance matrix computation with MEGA7(MUSCLE), MEGA7(CLUSTALW), FPP, and ML-DSP

DataSet	Parameter	MEGA7 (MUSCLE)	MEGA7 (CLUSTALW)	FFP	ML-DSP
Influenza Virus	Maximum Classification Accuracy	97.4%	97.4%	68.4%	100%
(38 sequences)	Average Classification Accuracy	93.4%	95.6%	57.0%	94.7%
Average Length: 1407bp	Processing Time	7.44 sec	2 min 14 sec	0.2 sec	0.2 sec
Mammalia	Maximum Classification Accuracy	95.1%	95.1%	49.6%	92.7%
(41 sequences)	Average Classification Accuracy	89.7%	90.7%	41.5%	87.8%
Average Length: 16647bp	Processing Time	11 min 15sec	5 hr 38 min	0.3 sec	0.3 sec
Vertebrates	Maximum Classification Accuracy	——	——	61.7%	99.7%
(4322 sequences)	Average Classification Accuracy	——	——	48.3%	98.3%
Average Length: 16806bp	Processing Time	>2 h	>6 h	94 sec	28 sec

As seen in Table 5 (columns 3, 4, and 6) ML-DSP overwhelmingly outperforms the alignment-based software MEGA7(MUSCLE/CLUSTALW) in terms of processing time. In terms of accuracy, for the smaller virus and mammalian benchmark datasets, the average accuracies of ML-DSP and MEGA7(MUSCLE/CLUSTALW) were comparable, probably due to the small size of the training set for ML-DSP. The advantage of ML-DSP over the alignment-based tools became more apparent for the larger vertebrate dataset, where the accuracies of ML-DSP and the alignment-based tools could not even be compared, as the alignment-based tools were so slow that they had to be terminated. In contrast, ML-DSP classified the entire set of 4322 vertebrate mtDNA genomes in 28 s, with average classification accuracy 98.3%. This indicates that ML-DSP is significantly more scalable than the alignment-based MEGA7(MUSCLE/CLUSTALW), as it can speedily and accurately classify datasets which alignment-based tools cannot even process.

As seen in Table 5 (columns 5 and 6), ML-DSP significantly outperforms the alignment-free software FFP in terms of accuracy (average classification accuracy 98.3% for ML-DSP vs. 48.3% for FFP, for the large vertebrate dataset), while at the same time being overall faster.

This comparison also indicates that, for these datasets, both alignment-free methods (ML-DSP and FFP) have an overwhelming advantage over the alignment-based methods (MEGA7 (MUSCLE/CLUSTALW)) in terms of processing time. Furthermore, when comparing the two alignment-free methods with each other, ML-DSP significantly outperforms FFP in terms of classification accuracy.

**Fig. 7:**
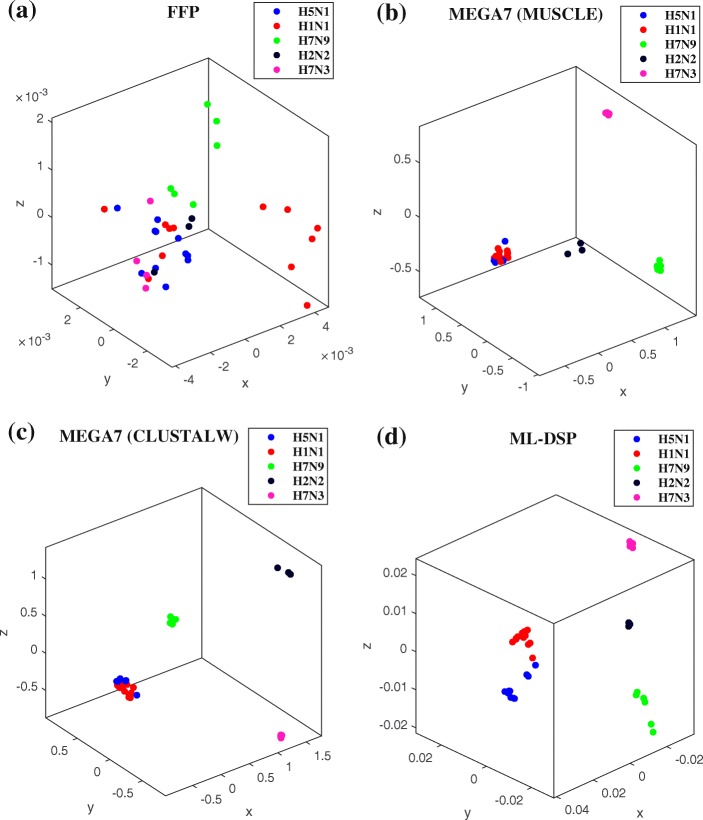
MoDMaps of the influenza virus dataset from Table 5, based on the four methods. The points represent viral genomes of subtypes H1N1 (red, 13 genomes), H2N2 (black, 3 genomes), H5N1 (blue, 11 genomes), H7N3 (magenta, 5 genomes), H7N9 (green, 6 genomes); ModMaps are generated using distance matrices computed with (**a**) FFP; (**b**) MEGA7(MUSCLE); (**c**) MEGA7(CLUSTALW); (**d**) ML-DSP

As another angle of comparison, Fig. 7 displays the MoDMaps of the first benchmark dataset (38 influenza virus genomes) produced from the distance matrices generated by FFP, MEGA7 (MUSCLE), MEGA7 (CLUSTALW), and ML-DSP respectively. Figure 7a shows that with FFP it is difficult to observe any visual separation of the dataset into subtype clusters. Figure 7b, MEGA7 (MUSCLE), and Fig. 7c MEGA7 (CLUSTALW) show overlaps of the clusters of points representing subtypes H1N1 and H2N2. In contrast, Fig. 7d, which visualizes the distance matrix produced by ML-DSP, shows a clear separation among all subtypes.

**Fig. 8:**
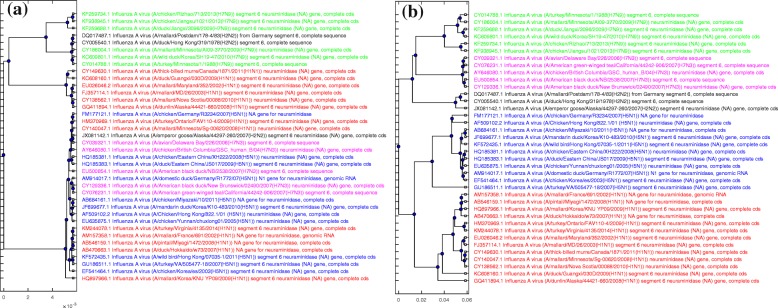
Phylogenetic tree comparison: FFP with ML-DSP. The phylogenetic tree generated for 38 influenza virus genomes using (**a**): FFP (**b**): ML-DSP

**Fig. 9:**
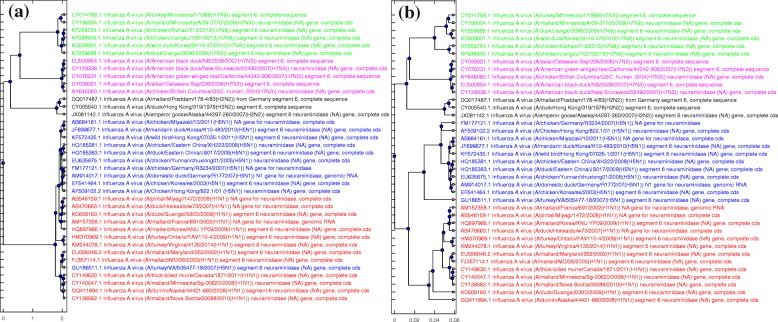
Phylogenetic tree comparison: MEGA7(MUSCLE/CLUSTALW) with ML-DSP. The phylogenetic tree generated for 38 influenza virus genomes using (**a**): MEGA7(MUSCLE/CLUSTALW) (**b**): ML-DSP

Finally Figs. 8 and 9 display the phylogenetic trees generated by each of the four methods considered. Figure 8a, the tree generated by FFP, has many misclassified genomes, which was expected given the MoDMap visualization of its distance matrix in Fig. 7a. Figure 9a displays the phylogenetic tree generated by MEGA7, which was the same for both MUSCLE and CLUSTALW: It has only one incorrectly classified H5N1 genome, placed in middle of H1N1 genomes. Figures 8b and 9b display the phylogenetic tree generated using the distance produced by ML-DSP (shown twice, in parallel with the other trees, for ease of comparison). ML-DSP classified all genomes correctly.

### Discussion

The computational efficiency of ML-DSP is due to the fact that it is alignment-free (hence it does not need multiple sequence alignment), while the combination of 1D numerical representations, Discrete Fourier Transform and Pearson Correlation Coefficient makes it extremely computationally time efficient, and thus scalable.

ML-DSP is not without limitations. We anticipate that the need for equal length sequences and use of length normalization could introduce issues with examination of small fragments of larger genome sequences. Usually genomes vary in length and thus length normalization always results in adding (up-sampling) or losing (down-sampling) some information. Although the Pearson Correlation Coefficient can distinguish the signal patterns even in small sequence fragments, and we did not find any considerable disadvantage while considering complete mitochondrial DNA genomes with their inevitable length variations, length normalization may cause issues when we deal with the fragments of genomes, and the much larger nuclear genome sequences.

Lastly, ML-DSP has two drawbacks, inherent in any supervised machine learning algorithm. The first is that ML-DSP is a black-box method which, while producing a highly accurate classification prediction, does not offer a (biological) explanation for its output. The second is that it relies on the existence of a training set from which it draws its “knowledge”, that is, a set consisting of known genomic sequences and their taxonomic labels. ML-DSP uses such a training set to “learn” how to classify new sequences into one of the taxonomic classes that it was trained on, but it is not able to assign it to a taxon that it has not been exposed to.

## Conclusions

We proposed ML-DSP, an ultrafast and accurate alignment-free supervised machine learning classification method based on digital signal processing of DNA sequences (and its software implementation). ML-DSP successfully addresses the limitations of alignment-free methods identified in [7], as follows:

(i) Lack of software implementation: ML-DSP is supplemented with freely available source-code. The ML-DSP software can be used with the provided datasets or any other custom dataset and provides the user with any (or all) of: pairwise distances, 3D sequence interrelationship visualization, phylogenetic trees, or classification accuracy scores. A quantitative comparison showed that ML-DSP significantly outperforms state-of-the-art alignment-based MEGA7 (MUSCLE/CLUSTALW) and alignment-free (FFP) software in terms of speed and classification accuracy.
(ii) Use of simulated sequences or very small real-world datasets: ML-DSP was successfully tested on a variety of large real-world datasets, comprising thousands of complete genomes, such as all complete mitochondrial DNA sequences available on NCBI at the time of this study, and similarly large sets of viral genomes and bacterial genomes. ML-DSP was tested in different evolutionary scenarios such as different levels of taxonomy (from domain to genus), small dataset (38 sequences), large dataset (4322 sequences), short sequences (1,136 bp), long sequences (1,999,595 bp), benchmark datasets of influenza virus and mammalian mtDNA genomes etc.
(iii) Memory overhead: ML-DSP uses neither *k*-mers nor any compression algorithms. Thus, scalability does not cause an exponential memory overhead, and a high classification accuracy is preserved with large datasets.

In addition, we provided a comprehensive quantitative analysis of all 13 one-dimensional numerical representations of DNA sequences used in the Genomic Signal Processing literature and found that, on average, the “PP”, “Just-A”, and “Real” representations performed better than others. We also showed that the classification accuracy of ML-DSP was significantly higher than the corresponding MoDMap visualizations of the dataset would indicate, likely due to the inherent dimensionality limitations of the latter. Lastly, we showed the potential for ML-DSP to be used for classifications of other DNA sequence genomic datasets, such as large datasets of complete viral or bacterial genomes.

## Availability and Requirements

**Project name:** ML-DSP

**Project home page:** https://github.com/grandhawa/MLDSP

**Operating system(s)**: Microsoft Windows

**Programming language:** MATLAB R2017A, license no. 964054

**License:** Creative Commons Attribution License

**Any restrictions to use by non-academics:** MATLAB license required

## Abbreviations

DFT: Discrete Fourier transform
DSP: Digital signal processing
FFP: Feature frequency profile
GSP: Genomic signal processing
MDS: Classical multidimensional scaling
MoDMap: Molecular distance map
PCC: Pearson correlation coefficient
PP: Purine/Pyrimidine
